# Receptor Model Source Apportionment of Nonmethane Hydrocarbons in Mexico City

**DOI:** 10.1100/tsw.2002.147

**Published:** 2002-03-29

**Authors:** V. Mugica, J. Watson, E. Vega, E. Reyes, M.E. Ruiz, J. Chow

**Affiliations:** Universidad Autónoma Metropolitana-Azcapotzalco. Av. San Pablo No. 180, Col. Reynosa Tamaulipas Azcapotzalco, 2200 México, DF., Mexico; Desert Research Institute, Reno, NV, USA; Instituto Mexicano del Petróleo, México D.F., Mexico

**Keywords:** air pollution, CMB model, Mexico City, source apportionment, VOC emissions

## Abstract

With the purpose of estimating the source contributions of nonmethane hydrocarbons (NMHC) to the atmosphere at three different sites in the Mexico City Metropolitan Area, 92 ambient air samples were measured from February 23 to March 22 of 1997. Light- and heavy-duty vehicular profiles were determined to differentiate the NMHC contribution of diesel and gasoline to the atmosphere. Food cooking source profiles were also determined for chemical mass balance receptor model application. Initial source contribution estimates were carried out to determine the adequate combination of source profiles and fitting species. Ambient samples of NMHC were apportioned to motor vehicle exhaust, gasoline vapor, handling and distribution of liquefied petroleum gas (LP gas), asphalt operations, painting operations, landfills, and food cooking. Both gasoline and diesel motor vehicle exhaust were the major NMHC contributors for all sites and times, with a percentage of up to 75%. The average motor vehicle exhaust contributions increased during the day. In contrast, LP gas contribution was higher during the morning than in the afternoon. Apportionment for the most abundant individual NMHC showed that the vehicular source is the major contributor to acetylene, ethylene, pentanes, n-hexane, toluene, and xylenes, while handling and distribution of LP gas was the major source contributor to propane and butanes. Comparison between CMB estimates of NMHC and the emission inventory showed a good agreement for vehicles, handling and distribution of LP gas, and painting operations; nevertheless, emissions from diesel exhaust and asphalt operations showed differences, and the results suggest that these emissions could be underestimated.

## INTRODUCTION

The Mexico City Metropolitan Area (MCMA), one of the biggest population concentrations in the world, lies at an altitude of 2,240 m above sea level. It is a basin with about 20 million inhabitants and an area of approximately 1,300 $km^2$. MCMA faces severe air quality problems due to the fact that standards are exceeded throughout most of the year. Ozone and other photochemical oxidants are produced in the atmosphere through a complex sequence of reactions involving nonmethane hydrocarbons (NMHC) and nitrogen oxides[1]. The knowledge of the composition, amount, and sources of NMHC emissions is essential to develop cost-effective abatement strategies to achieve a reduction of ozone levels. The chemical mass balance (CMB) is one of the receptor models that have been applied to air resource management. Receptor models use the chemical and physical characteristics of particles and gases, measured in both source and receptor, to quantify source contributions to receptor concentration[2]. The CMB model has been applied in different countries to the apportionment of NMHC to their source types[3,4,5,6] and also to validate emission inventories[7,8]. The CMB model requires speciated measurements of ambient NMHC at one or more receptor sites, as well as detailed characterization of the emission profiles from the most important sources of pollutants. Fujita[9] applied the CMB receptor model in El Paso del Norte and the City of Juarez at the border of Mexico and the U.S., using profiles developed in the U.S. Studies have been conducted to determine the concentrations of NMHC in the MCMA atmosphere[10], and different NMHC source profiles have been developed[11,12,13]. Vega et al.[14] applied the CMB receptor model using ambient NMHC data and source profiles developed in Mexico City in 1996. By the end of 1996, the gasoline and fuel oil composition changed because of new regulations. A new gasoline known as Premium, with a higher content of substituted paraffins, was introduced. The leaded gasoline Nova was eliminated at the end of 1997, and the benzene content was reduced in Magna gasoline[15]. As a consequence, the chemical composition of vehicular emissions changed.

The main objective of this paper is the application of the CMB model, based on the ambient data obtained in 1997, to estimate NMHC source contributions. New vehicular profiles for light- and heavy-duty vehicles were determined in Mexico City with the purpose of resolving gasoline and diesel vehicle exhaust NMHC contributions. Food cooking profiles were developed to allow a better understanding of the high levels of LP gas components (propane and butane) in Mexico City's atmosphere. Apportionment of the most abundant NMHC species was estimated and the comparison of NMHC source contribution during the morning and afternoon was carried out for the first time.

## METHODS

### Ambient Air Sampling

In 1997 a NMHC sampling campaign was carried out according to the EPA protocol TO-14[16]; 6-h samples (6:00 a.m. to 12:00 p.m. and 12:00 p.m. to 6:00 p.m.) were collected in 6-l SUMMA® stainless steel canisters from February 23 to March 22 in the center of the city (La Merced station). In addition, 3-h samples (6:00 to 9:00 a.m.) were collected from March 11 to 23 in the northeast, center, and southwest of the city (Fig. 1). These sites were selected because they represent different types of land use. Xalostoc, at the northeast, is an industrial area that has heavy traffic. La Merced is a commercial area near downtown, with markets, restaurants, and important avenues heavily traveled by both gasoline and diesel vehicles. Pedregal, at the southwest part of the city, is a high-income neighborhood with lightly traveled roads. During the

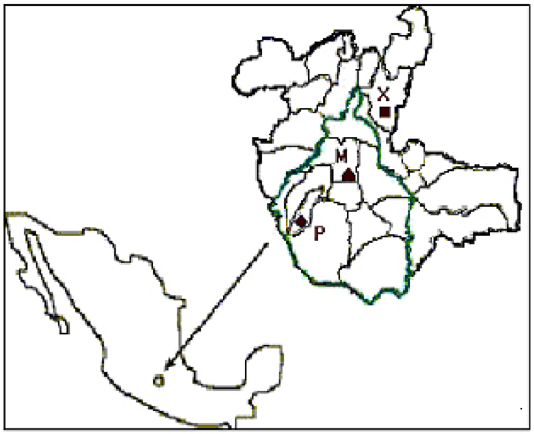

**FIGURE 1.** Sampling sites: Xalostoc (X), Merced (M), and Pedregal (P).

field campaign, meteorological observations registered that wind flows were very light. Winds in the well-mixed boundary layers showed significant changes in direction; therefore, the use of surface wind data alone to determine pollutant dispersion patterns may be very misleading. Although recirculation of pollutants within the course of a day appeared to be important, recirculation over several days did not result in pollutant buildup during the period of study[17].

The uncertainties for each compound concentration were estimated with the following equation, which has been used in other studies[3,7]:

$$\sigma(c) = [(2 \times MDL)^2 + (CV \times c)^2]^{1/2}$$

where $\sigma(c)$ is the root mean square error for the concentration value ($c$), $MDL$ is the minimum detection limit for the GC-FID (gas chromatography with flame ionization detection) method (0.2 ppbC), and $CV$ is the coefficient of variation ( ± 10%). The uncertainties are explicit inputs to the CMB receptor model. Ambient data near the detection limit is automatically given less weight in the CMB calculations because the equation assigns higher uncertainties to these values.

## Source Profiles

Different source types of NMHC were selected considering the emission inventory for MCMA. In addition to source profiles previously developed, new source profiles were obtained for CMB model application. In this study the source profiles represent the fractional amount of every species of the total NMHC emissions from each source type.

## Vehicular Profiles

Resolving contributions from diesel- and gasoline-powered vehicles is a major challenge for researchers in the air quality field. Therefore, to distinguish diesel and gasoline emissions, two tunnels, three crossroads, one bus station, and two truck stations were selected for the sampling of motor vehicle exhaust. Due to the differences in the fuel usage of each setting, a total of 36 samples were obtained. The average integrated sampling time for the settings lasted 2 h in each case[12]. These sampling campaigns were carried out during the winter of 1998.

The first tunnel (Insurgentes) is 365 m long, 7.77 m wide, and 4.30 m high. It is located near the downtown area (EXHTI), and is used mostly by gasoline-powered vehicles. The second tunnel

(EXHTN) is 280 m long, 10 m wide, and 9 m high, and it is located in an industrial district to the northwest of the city (Naucalpan), trafficked by both gasoline- and diesel-powered vehicles (78 and 22%, respectively). Three crossroads were selected, each with a different proportion between gasoline- and diesel-powered vehicle transit; the first site is close to the Insurgentes tunnel; some buses drove through, but the majority of vehicles were gasoline powered. The second crossroad is 300 m away from the Naucalpan tunnel exit. In this crossroad there are four avenues; the number of vehicles was three times larger than in the first crossroad, consisting of 87% gasoline-powered and 13% diesel-powered vehicles[18]. The last crossroad is located in the Xalostoc industrial zone, which was trafficked by 74% gasoline-powered and 26% diesel-powered vehicles. The profile EXHC was developed according to the average of all samples collected at crossroads.

Three different sites were selected to determine the profiles of diesel-powered vehicles: one bus terminal and two truck terminals. The bus terminal is located at the northeast of the MCMA and has several avenues surrounding it. Sampling was carried out at two different bus terminal exits. Bus transit was constant and abundant during the sampling periods. In the first truck terminal, there was constant vehicle traffic during the sampling time at both entrances. It was estimated that around 10% of the vehicles were gasoline-powered trucks. The second truck terminal is located at the northeast of the city, where avenues and industries surround the zone. Almost 90% of the trucks there were diesel powered. The diesel-powered vehicle source profile (EXHDIE) was developed with the average of 19 samples taken in these three sites[12].

## Food Cooking Profiles

Gaseous streams from two restaurants with charcoal grills and LP gas stoves, two *tortillerías,* food fryers, and LP gas rotisseries were sampled using 6-l stainless steel canisters for further analysis of NMHC by GC-FID[13]. Two profiles were used in this study. The first was obtained from the average of the restaurant samples composition (FOODR), and the second from the fried food profile (FOOD), since in this type of restaurant a different kind of food is prepared.

## Other Profiles

Profiles determined previously in Mexico City from architectural coating, asphalt operations, whole Magna gasoline, hot soak, LP gas, and sanitary landfill were used in this study[14]. To develop the average emission profile of coatings and graphic arts, samples of different processes such as architectonic coating (vinyl and acrylic), applications of lacquers, varnishes, automotive paints, serigraphy, and offset were taken. A road where slow-curing asphalt pavement application was taking place was selected to obtain five duplicated samples of this source profile. Five samples were collected directly from the landfill's line pipes. The average composition of 13 samples from 4 different vendors of liquefied petroleum gas in liquid phase (LP gas) was used to calculate the LPG vapor compositions by means of the Rayleigh's equation. Different samples of Magna gasoline were purchased and analyzed to determine the chemical compositions in order to conform the profiles. The profile of whole Premium gasoline introduced at the end of 1996 was determined in this study by the composition analysis of five samples purchased in different gas stations. NMHC, other than the 61 selected species in the ambient database, and those reported as "unidentified" were grouped to get a source profile, which corresponds to the "others" source. Table 1 lists the source types, an identifier, and a brief description of the source profiles used for source apportionment of the NMHC measurements.

**TABLE 1**
**Description of Source Profiles**

| Source Type | Identification | Description |
|---|---|---|
| Vehicular exhaust | EXHTI[12] | Insurgentes tunnel profile; light-duty vehicles (99%) |
| | EXHTN[12] | Naucalpan tunnel profile; light-duty vehicles (78%), heavy-duty vehicles (22%) |
| | EXHC[12] | Average of three crossroads profiles |
| | EXHDIE[12] | Average of exhaust, heavy-duty vehicles |
| Fuel evaporation | HOTS[14] | Hot soak profile (gasoline vapor) |
| Whole gasoline | WGAM[14] | Whole Magna gasoline |
| | WGASP[12] | Whole Premium gasoline |
| LP gas | LPG[14] | Liquefied petroleum gas (gas phase) |
| Food | FOODR[13] | Average of source profiles from restaurants |
| | FOOD[13] | Source profile from fried food restaurants |
| Asphalt | ASPHA[14] | Asphalt plant and asphalt operations |
| Landfill | LAFIL[14] | Emissions from landfill |
| Solvent | VINPA[14] | Acrylic and vinyl architectural paints |
| Others | OTHR | Sum of species unidentified and not considered |

Table 2 shows the source profiles used in this study. The profiles are expressed as parts per billion of carbon percentage (ppbC%) of total NMHC. The source profile uncertainties were calculated as the standard deviations from averaging the results of the different samplings of each source and their replicates.

Because of NMHC decay through photooxidation mechanisms, principally by reaction with the OH-radical, the source profile pattern may be distorted as a plume moves from source to receptor[3]. The lifetimes of different NMHC were examined to determine which compounds might retain their relative abundance between source and receptor and which would not in order to select the fitting species (marked with an asterisk in Table 2)[19]. These species are major constituents in all samples and have atmospheric lifetimes equal to or greater than that of toluene. Olefinic NMHC are usually very reactive and thus were excluded as fitting species. Although the reactive species were not used to calculate source contributions, they were retained as floating species because the comparison of calculated and measured values for floating species is part of the model validation process[20].

## Analysis

Samples were analyzed for full speciation by GC-FID after a cryogenic sample concentration in a freeze-out loop made from chromatographic-grade stainless steel tubing packed with 60/80 mesh deactivated glass beads. A separate GC-FID with preconcentration system was used for speciation of two carbon compounds. In this paper, NMHC refers to all FID chromato-graphic peaks (including unidentified) of molecular weight compounds up to *n*-undecane.

## Model Application

The CMB model relates speciated measurements of ambient NMHC with the compositions of the NMHC from different source categories in order to determine the contribution of each source modelled. In comparison with other source-based models, the CMB is useful because it does not depend on emission inventory data, which can be unreliable. The contributions are determined by a least-square solution to a set of linear equations that express each receptor concentration of a chemical species as a linear sum of products of source profile species of NMHC and source

**TABLE 2**
**Source Profiles for MCMA**

| | EXHTI | EXHTN | EXHC | EXHDIE | HOTS | WGAM | WGASP | FOOD | FOODR | LPG | VINPA | ASPHA | LAFIL | OTHR |
|---|---|---|---|---|---|---|---|---|---|---|---|---|---|---|
| Ethane* | 1.18 | 0.62 | 0.62 | 0.60 | 0.11 | 0.00 | 0.00 | 1.24 | 4.11 | 0.98 | 0.00 | 0.50 | 0.00 | |
| Ethylene | 3.55 | 3.01 | 3.05 | 2.11 | 0.49 | 0.00 | 0.00 | 9.20 | 8.04 | 0.00 | 0.00 | 2.30 | 0.46 | |
| Acetylene* | 8.01 | 5.78 | 5.11 | 2.23 | 0.44 | 0.00 | 0.00 | 6.37 | 5.21 | 0.00 | 0.00 | 3.24 | 0.90 | |
| Propane* | 3.48 | 5.94 | 6.93 | 6.56 | 0.37 | 0.04 | 0.00 | 2.92 | 21.45 | 67.66 | 0.00 | 10.5 | 4.77 | |
| Propene | 1.95 | 1.58 | 1.73 | 1.42 | 0.00 | 0.00 | 0.00 | 30.00 | 2.77 | 0.22 | 0.00 | 0.99 | 0.90 | |
| i-Butane* | 0.98 | 1.51 | 1.51 | 2.07 | 0.83 | 0.28 | 0.19 | 6.23 | 5.09 | 12.07 | 0.00 | 2.61 | 1.31 | |
| 1-Butene | 0.00 | 0.00 | 0.00 | 0.11 | 0.13 | 0.16 | 0.04 | 0.00 | 0.00 | 0.81 | 0.05 | 0.86 | 0.00 | |
| n-Butane* | 3.11 | 4.32 | 4.47 | 4.35 | 3.63 | 2.40 | 2.95 | 16.62 | 14.40 | 16.30 | 0.01 | 5.77 | 3.46 | |
| t-2Butene | 0.27 | 0.18 | 0.24 | 0.15 | 0.70 | 0.23 | 0.08 | 0.11 | 0.20 | 0.72 | 0.00 | 0.00 | 0.00 | |
| c-2Butene | 0.23 | 0.19 | 0.21 | 0.15 | 0.63 | 0.23 | 0.08 | 0.10 | 0.13 | 0.47 | 0.00 | 0.00 | 0.00 | |
| 3Me1Butene | 0.43 | 0.69 | 0.56 | 0.53 | 0.27 | 0.09 | 0.07 | 0.00 | 0.00 | 0.00 | 0.01 | 0.69 | 0.00 | |
| i-Pentane* | 7.07 | 7.28 | 7.86 | 8.24 | 18.67 | 9.06 | 6.85 | 2.85 | 2.38 | 0.14 | 0.03 | 1.74 | 2.92 | |
| 1-Pentene | 0.22 | 0.20 | 0.22 | 0.12 | 0.59 | 0.29 | 0.18 | 0.07 | 0.17 | 0.00 | 0.00 | 0.00 | 0.00 | |
| 2Me1Butene | 0.39 | 0.34 | 0.41 | 0.38 | 0.61 | 0.44 | 0.44 | 0.14 | 0.14 | 0.00 | 0.00 | 0.28 | 0.00 | |
| n-Pentane* | 2.69 | 2.90 | 2.90 | 1.98 | 11.49 | 4.82 | 1.85 | 2.93 | 0.97 | 0.02 | 0.00 | 1.48 | 1.55 | |
| Isoprene | 0.67 | 0.85 | 0.00 | 0.00 | 0.07 | 0.00 | 0.00 | 0.00 | 0.00 | 0.00 | 0.00 | 0.00 | 0.00 | |
| t-2Pentene | 0.00 | 0.00 | 0.42 | 0.28 | 1.33 | 0.68 | 0.61 | 0.00 | 0.00 | 0.00 | 0.00 | 0.00 | 0.00 | |
| c-2Pentene | 0.00 | 0.00 | 0.00 | 0.09 | 0.70 | 0.35 | 0.30 | 0.00 | 0.00 | 0.00 | 0.00 | 0.00 | 0.00 | |
| 2Me2Butene | 0.55 | 0.49 | 0.52 | 0.39 | 1.37 | 0.83 | 0.83 | 0.14 | 0.13 | 0.00 | 0.00 | 0.00 | 0.00 | |
| MTBE | 3.77 | 4.35 | 4.18 | 3.89 | 8.56 | 4.75 | 4.70 | 0.94 | 0.56 | 0.00 | 0.00 | 1.29 | 0.95 | |
| 2,2DMButane* | 0.14 | 0.00 | 0.89 | 0.94 | 1.02 | 0.64 | 0.19 | 0.21 | 0.20 | 0.00 | 0.00 | 0.22 | 0.00 | |
| CycloPentene | 0.22 | 0.20 | 0.31 | 0.32 | 0.26 | 0.11 | 0.13 | 0.17 | 0.30 | 0.00 | 0.00 | 0.00 | 0.00 | |
| 4Me1Pentene | 0.00 | 0.00 | 0.42 | 0.28 | 1.33 | 0.68 | 0.61 | 0.00 | 0.00 | 0.00 | 0.00 | 0.00 | 0.00 | |
| 2Me1Pentene | 0.00 | 0.00 | 0.00 | 0.09 | 0.70 | 0.35 | 0.30 | 0.05 | 0.00 | 0.00 | 0.00 | 0.00 | 0.00 | |
| CycloPentane* | 0.00 | 0.00 | 0.00 | 0.00 | 0.80 | 0.39 | 9.58 | 0.00 | 0.00 | 0.00 | 0.00 | 0.00 | 0.00 | |
| 2MePentane* | 2.70 | 2.93 | 2.87 | 2.40 | 5.86 | 3.89 | 2.10 | 0.67 | 0.47 | 0.00 | 0.07 | 1.40 | 1.15 | |
| 3MePentane* | 1.61 | 1.75 | 1.73 | 2.74 | 3.26 | 0.00 | 1.23 | 0.38 | 0.40 | 0.00 | 0.07 | 0.00 | 0.00 | |
| n-Hexane* | 2.11 | 2.32 | 2.16 | 2.68 | 4.18 | 2.43 | 0.90 | 0.85 | 0.94 | 0.00 | 0.08 | 2.13 | 0.95 | |
| t2Hexene | 0.04 | 0.02 | 0.02 | 0.00 | 0.15 | 0.00 | 0.04 | 0.01 | 0.00 | 0.00 | 0.00 | 0.00 | 0.00 | |
| c2Hexene | 0.14 | 0.18 | 0.19 | 0.28 | 0.37 | 0.00 | 0.13 | 0.00 | 0.41 | 0.00 | 0.00 | 0.00 | 0.00 | |
| Perchlethylene* | 0.00 | 0.06 | 0.01 | 0.38 | 0.30 | 0.00 | 0.00 | 0.00 | 0.02 | 0.00 | 0.00 | 0.00 | 0.00 | |
| McyPentane* | 0.09 | 0.11 | 0.10 | 0.21 | 1.32 | 1.08 | 0.54 | 0.01 | 0.01 | 0.00 | 0.00 | 0.32 | 0.35 | |
| 24DMPentane* | 0.66 | 0.73 | 0.67 | 0.38 | 0.59 | 0.75 | 2.52 | 0.14 | 0.06 | 0.00 | 0.00 | 0.21 | 0.00 | |
| Benzene* | 2.43 | 2.12 | 2.26 | 1.71 | 1.89 | 1.13 | 1.46 | 0.70 | 3.19 | 0.00 | 0.01 | 0.69 | 0.68 | |
| CycloHexane* | 0.77 | 0.82 | 0.76 | 0.18 | 0.36 | 0.20 | 0.09 | 0.18 | 0.21 | 0.00 | 0.00 | 0.00 | 0.00 | |
| 2Mhexane* | 0.89 | 0.95 | 0.91 | 0.59 | 1.02 | 1.96 | 0.68 | 0.21 | 0.14 | 0.00 | 0.00 | 0.49 | 0.48 | |
| 23DMPentane* | 0.79 | 0.85 | 0.79 | 0.36 | 0.53 | 0.00 | 3.98 | 0.17 | 0.09 | 0.00 | 0.00 | 0.28 | 0.00 | |
| 3MeHexane* | 0.98 | 1.08 | 1.04 | 0.75 | 1.04 | 1.28 | 0.70 | 0.44 | 0.25 | 0.00 | 0.00 | 0.68 | 0.67 | |
| 224TMPentane | 3.90 | 4.24 | 4.05 | 0.28 | 3.17 | 6.02 | 14.49 | 0.81 | 0.44 | 0.00 | 0.00 | 1.10 | 1.00 | |
| n-Heptane* | 0.90 | 0.88 | 0.88 | 0.62 | 0.95 | 1.17 | 0.44 | 1.40 | 0.33 | 0.00 | 0.00 | 0.46 | 0.50 | |
| MeCyHexane* | 0.41 | 0.38 | 0.33 | 0.16 | 0.44 | 0.48 | 0.20 | 0.06 | 0.01 | 0.00 | 0.00 | 0.23 | 0.00 | |
| 234TMPentane | 1.55 | 1.68 | 1.60 | 0.30 | 1.23 | 3.29 | 6.08 | 0.29 | 0.15 | 0.00 | 0.00 | 0.50 | 0.35 | |
| Toluene* | 6.75 | 7.16 | 7.33 | 8.64 | 3.23 | 7.45 | 5.19 | 1.76 | 3.88 | 0.00 | 0.41 | 6.76 | 4.28 | |
| 2MeHepatane | 0.43 | 0.37 | 0.37 | 0.19 | 0.29 | 0.55 | 0.22 | 0.01 | 0.01 | 0.00 | 0.00 | 0.20 | 0.00 | |
| 3MeHeptane* | 0.45 | 0.43 | 0.42 | 0.19 | 0.13 | 0.64 | 0.11 | 0.01 | 0.02 | 0.00 | 0.00 | 0.22 | 0.00 | |
| 225TMHexane | 0.00 | 0.00 | 0.00 | 0.02 | 0.02 | 0.00 | 1.44 | 0.00 | 0.00 | 0.00 | 0.00 | 0.00 | 0.00 | |
| n-Octane* | 0.59 | 0.45 | 0.46 | 0.30 | 0.35 | 0.78 | 0.14 | 1.34 | 0.24 | 0.00 | 0.00 | 0.46 | 0.38 | |
| 25dm-Heptane | 0.09 | 0.06 | 0.00 | 0.00 | 0.13 | 0.00 | 0.04 | 0.00 | 0.00 | 0.00 | 0.00 | 0.00 | 0.00 | |
| EtBenzene | 1.41 | 1.34 | 1.52 | 2.61 | 0.61 | 1.33 | 1.11 | 0.51 | 0.73 | 0.00 | 0.02 | 3.68 | 6.92 | |
| M/pXylene | 4.87 | 4.35 | 5.22 | 10.68 | 2.49 | 4.67 | 3.38 | 1.85 | 2.74 | 0.00 | 0.06 | 14.15 | 26.48 | |
| 3MeOctane | 0.31 | 0.21 | 0.23 | 0.20 | 0.16 | 0.33 | 0.08 | 0.01 | 0.00 | 0.00 | 0.00 | 0.00 | 0.00 | |
| Styrene | 0.27 | 0.23 | 0.27 | 0.38 | 0.05 | 0.00 | 0.00 | 0.08 | 0.23 | 0.00 | 0.00 | 0.26 | 0.28 | |
| o-Xylene | 1.88 | 1.65 | 1.96 | 3.85 | 0.93 | 2.43 | 1.38 | 0.66 | 1.09 | 0.00 | 0.02 | 4.38 | 9.74 | |
| n-Nonane* | 0.57 | 0.33 | 0.35 | 0.55 | 0.20 | 0.54 | 0.05 | 0.10 | 0.12 | 0.00 | 0.00 | 1.07 | 0.57 | |
| IpropBenzen | 0.18 | 0.15 | 0.15 | 0.14 | 0.07 | 0.14 | 0.07 | 0.01 | 0.00 | 0.00 | 0.00 | 0.23 | 0.00 | |
| n-PropBenzene | 0.37 | 0.36 | 0.34 | 0.24 | 0.16 | 0.51 | 0.22 | 0.06 | 0.06 | 0.00 | 0.00 | 0.46 | 0.00 | |
| 135TMBenz | 0.73 | 0.67 | 0.63 | 0.45 | 0.28 | 0.77 | 0.36 | 0.06 | 0.07 | 0.00 | 0.00 | 1.05 | 0.00 | |
| MetToluene | 1.25 | 1.25 | 1.16 | 0.56 | 0.50 | 1.52 | 0.00 | 0.15 | 0.18 | 0.00 | 0.00 | 0.86 | 0.61 | |
| 124TrMBenz | 0.31 | 0.28 | 0.27 | 0.68 | 0.66 | 2.23 | 1.12 | 0.06 | 0.12 | 0.00 | 0.00 | 1.57 | 1.07 | |
| n-Decane | 0.21 | 0.21 | 0.18 | 0.07 | 0.10 | 0.48 | 0.05 | 0.01 | 0.00 | 0.00 | 0.00 | 2.58 | 0.83 | |
| n-Undecane | 0.06 | 0.07 | 0.03 | 0.09 | 0.05 | 0.26 | 0.00 | 0.05 | 0.06 | 0.00 | 0.00 | 3.44 | 0.40 | |
| Unidentified | 6.59 | 6.75 | 5.15 | 0.06 | 0.00 | 0.00 | 0.18 | 5.80 | 11.00 | 0.00 | 0.03 | 0.30 | 5.28 | 100 |

contributions. The input data to the CMB model are the source profile species and the receptor concentrations, each with uncertainty estimates. The input data uncertainties are used both to weight the relative importance of the input data to the model solution and to estimate uncertainties of the source contributions. The output consists of the contributions for each source type to the total ambient NMHC as well as to individual NMHC concentrations. The base of the receptor model is the following equation, which expresses the relationship between the concentrations of the chemical species measured in the receptor with those emitted in the source.

$$C_i = \sum_{j=i}^{P} F_{ij} \bullet S_j$$

where $C_i$ is the ambient concentration of the species $i$ measured at the receptor site; $P$ is the number of sources that contribute; $F_{ij}$ is the fraction of source contribution $S_j$ composed of element $i$. The number of chemical species ($i$) must be greater or equal to the number of sources ($j$) for a unique solution to this equation[2].

The CMB model provides values for several performance measures to evaluate the solution. These measured values include chi-square ($\chi^2$), the weighted sum of the squared differences between calculated and measured fitting species concentrations divided by the effective variance and degrees of freedom (ideally $\chi^2$ would be zero, but values up to 4 are acceptable). $R^2$ is the fraction of the variance in the receptor concentrations. $R^2$ ranges from 0 to 1; when $R^2$ is less than 0.8 the source contribution estimates do not explain well the observations with the fitting source profiles[2].

The assumptions of the CMB model are: (1) composition of source emissions are constant over the period of ambient and source sampling, (2) chemical species do not react with each other, (3) all sources with potential for significant contribution to the receptor have been identified and have had their emission characterized, (4) the source compositions are linearly independent of each other, (5) the number of source categories is less than or equal to the number of chemical species, and (6) measurement uncertainties are random, uncorrelated, and normally distributed[4].

CMB software in current use is the CMB8, which operates in a Windows®-based environment and accepts inputs and creates outputs in a wider variety of formats than previous CMB versions. The software is interactive, allowing sensitivity and assumptions to be performed[21].

## Initial Tests

The CMB8 program was applied to La Merced morning ambient data (6:00 a.m. to 12:00 p.m.), following the methodology described by Fujita et al.[3]. Different combinations of source profiles and fitting species were used in order to determine the effects of alternative source profiles on the source contribution estimates. Initial tests were performed applying the CMB model considering only three sources: vehicular (EXHTI [tunnel profile] or EXHC [rossroads profile]), LP gas, and asphalt operations. When the EXHTI profile was substituted for the EXHC profile, the contribution of asphalt operations was the same, but the vehicular source contribution increased and the LP gas source decreased. The explanation is that the EXHC profile was determined using samples from the crossroads, which have some influence from the ambient air, and the vehicular profile includes emissions due to handling and distribution of LP gas, therefore the model attributes part of the LP gas emissions to the vehicular source.

Two initial tests were performed with the intention of verifying the consistency and stability of evaporative-, gasoline-, and diesel-exhaust source contributions. If the tunnel exhaust profile is the only one used, the total mass due to vehicular emissions is apportioned to the gasoline exhaust source. If the three profiles are used, it is possible to obtain the contributions from diesel exhaust and evaporative emissions, although most of the mass is due to gasoline exhaust emissions.

Both EXHTI and EXHTN provided good fits, but EXHTI was selected as the default gasoline exhaust profile because the tunnel in which the samples were taken was trafficked mostly by gasoline-powered vehicles, while more than 20% of the vehicles using the tunnel in which the EXHTN profile was developed were diesel-powered. In addition, when EXHTN was used as the gasoline profile, the contribution of diesel vehicular source (EXHDIE) was lower, because the EXHTN profile includes some of the diesel emissions. If the EXHTI profile is applied, there is a better identification of the two sources (gasoline- and diesel-powered vehicles).

When the whole liquid gasoline source profiles (Magna and Premium) were included simultaneously with the other vehicle profiles (exhaust and evaporative), some uncertainty/similarity clusters (collinearity) appeared and the fits were not consistent in all cases; therefore, the whole gasoline profiles were excluded. This collinearity could be due to the fact that the vehicle exhaust emissions have an important quantity of unburned fuel, and the exhaust profiles include part of these fuel emissions.

Other studies[14,22] have found an important contribution of NMHC from the handling and distribution of LP gas. This study claims that part of the LP gas emissions is originated during food preparation. This is the reason why emission profiles from food cooking were determined. When initial tests were performed including the food cooking profile in addition to the LP gas profile, some of the emissions attributed to the handling and distribution of LP gas were apportioned to food cooking emissions. At the same time, VINPA was used as the default profile to represent emissions from architectural coatings, varnishes, vehicle coatings, and graphic arts processes[14]. Dry cleaning and degreasing profiles were tested and their contributions were negligible.

As a result of the initial tests, the following default set of source profiles was applied to the CMB model: EXHTI, EXHDIE, HOTS, ASPHA, LPG, FOODR, VINPA, and LAFIL (Table 2).

## RESULTS AND DISCUSSION

### Model Performance

Model outputs (source contribution estimates and statistics) were individually examined for each CMB fit to determine the validity of model results. The parameters $R^2$ and $\chi^2$ calculated by the model were within the acceptable values. In Xalostoc, La Merced, and Pedregal, the average of $R^2$ was 0.95 at the three sites, and $\chi^2$ had average values of 2, 2.5, and 2.2, respectively. Calculated mass values were higher than 80% of measured mass in all cases. This is an indicator that the significant sources were included. Fig. 2 shows the scatterplots of all calculated vs. measured NMHC mass concentrations. In most cases the calculated and the measured mass of both fitting and floating species were similar, with a variation of less than 20%. Fig. 3 shows the

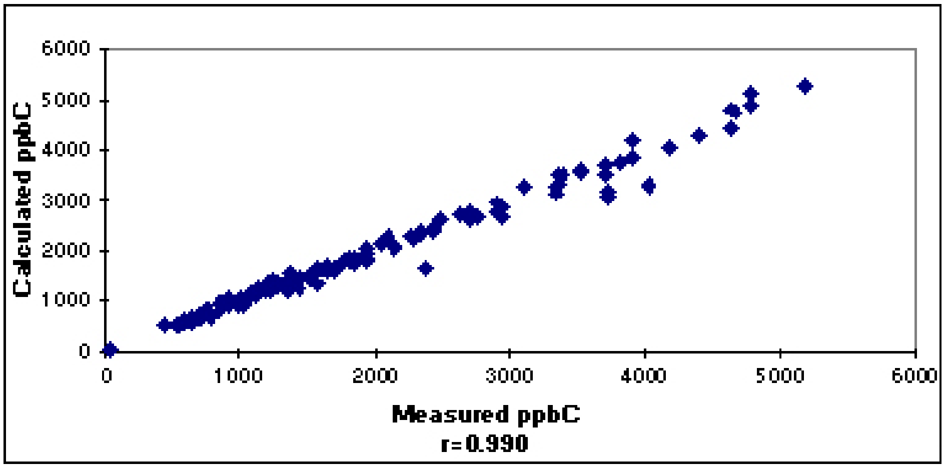

**FIGURE 2.** Measured vs. calculated NMHC total mass concentrations.

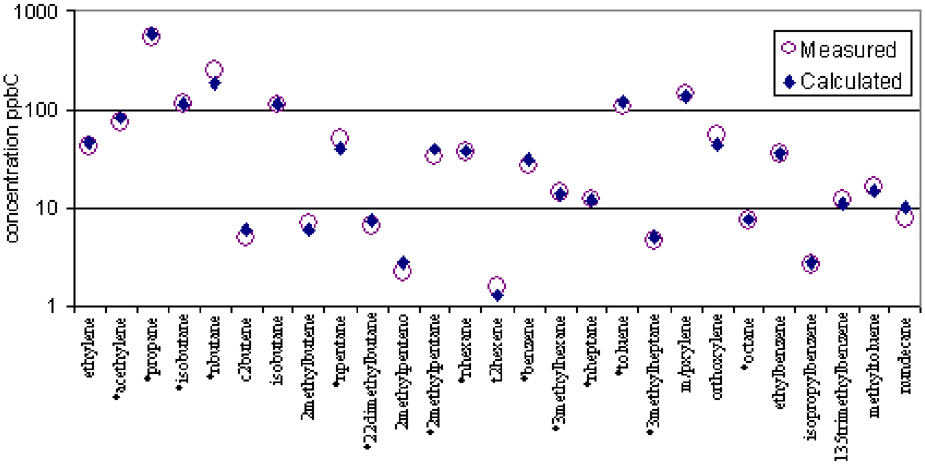

**FIGURE 3.** Comparison of the measured and calculated ambient concentrations of the mass balance fitting(*) and not fitting species used in the NMHC source apportionment for March 13, 1997.

agreement between the model-calculated mass balance compound concentrations and those measured at the Xalostoc site for March 13, 1997. Good agreement is observed between the calculated and measured concentrations of organic species for this site as well as for other sites. The deviations from CMB model assumptions were analyzed, with the finding that all of them were met for this study.

## Temporal Variation of CMB Apportionment in La Merced

In general, ambient air samples taken in La Merced station during the morning were twice as concentrated as those taken during the afternoon, with an average of 2,712 ± 1,092 ppbC vs. 1,211 ± 524 ppbC. The major contributors of NMHC in La Merced during the morning and afternoon are presented in Table 3. In both cases the major contributors to NMHC were the vehicular sources: gasoline-powered vehicles contributing an average of 45% (morning) and 48% (afternoon), and diesel-powered vehicles contributing 14% (morning) and 29% (afternoon). This is due to the fact that the number of diesel-powered vehicles is increased after 12:00 p.m., because the trucks circulate along the whole area. The evaporative emissions were greater during the morning (5.7%) than during the afternoon (2.4%).

The average contribution of LP gas in La Merced station during the morning was 12 ± 6%, which is lower compared to 20 ± 4% reported in other studies[14]. These results were obtained because some of these emissions were apportioned as the food cooking source. Emissions from the LP gas source diminished during the afternoon to an average of 4%. The results of the present study are in agreement with the conclusions of Blake and Rowland[22]. The food cooking contribution increased during the afternoon, due to the fact that in Mexico City the lunchtime is from 2:00 to 4:00 p.m. Asphalt operations had similar contribution percentages in both periods (14 ± 2 and 12 ± 2%). The landfill emissions were only apportioned during the morning for 5 days, with contributions up to 4%. The emissions due to painting operations were detected in percentages up to 3%, especially during the morning.

**TABLE 3**
**Source Contributions in La Merced**

**Morning: 6:00 a.m. to 12:00 p.m.**

| Date | Measured mass ppb C | Calculated mass % | Gasoline exhaust % | Diesel exhaust % | Evap % | LP gas % | Food cook. % | Asphalt % | Paint % | Land-fill % |
|---|---|---|---|---|---|---|---|---|---|---|
| 23-02 | 1574 | 106.3 | 49 | 9 | 9 | 22 | 2 | 15 | 0 | 0 |
| 24-02 | 3115 | 105.6 | 56 | 5 | 10 | 18 | 4 | 13 | 0 | 0 |
| 25-02 | 3395 | 104.3 | 47 | 9 | 13 | 13 | 9 | 14 | 0 | 0 |
| 26-02 | 2427 | 101 | 39 | 21 | 10 | 8 | 13 | 10 | 0 | 0 |
| 27-02 | 2915 | 100.9 | 37 | 19 | 13 | 11 | 8 | 13 | 0 | 0 |
| 28-02 | 3361 | 105 | 48 | 14 | 6 | 19 | 4 | 13 | 1 | 0 |
| 01-03 | 3335 | 98.1 | 41 | 13 | 7 | 20 | 4 | 12 | 1 | 0 |
| 02-03 | 2697 | 102.1 | 35 | 13 | 7 | 21 | 12 | 14 | 0 | 0 |
| 03-03 | 2148 | 98.3 | 43 | 12 | 8 | 13 | 8 | 14 | 0 | 0 |
| 04-03 | 4793 | 107 | 47 | 14 | 7 | 17 | 5 | 14 | 3 | 0 |
| 05-03 | 3897 | 107 | 38 | 27 | 7 | 10 | 10 | 15 | 0 | 0 |
| 06-03 | 698 | 95.8 | 51 | 13 | 7 | 7 | 4 | 13 | 0.8 | 0 |
| 07-03 | 1646 | 98 | 50 | 12 | 5 | 10 | 4 | 16 | 0 | 1 |
| 08-03 | 1695 | 98 | 46 | 15 | 2 | 12 | 6 | 13 | 0 | 4 |
| 09-03 | 1309 | 98 | 44 | 12 | 1 | 19 | 8 | 13 | 1 | 0 |
| 10-03 | 1849 | 99.7 | 46 | 16 | 5 | 10 | 7 | 16 | 0 | 0 |
| 11-03 | 3713 | 95 | 44 | 14 | 6 | 9 | 9 | 12 | 0 | 1 |
| 12-03 | 3529 | 103 | 51 | 9 | 6 | 12 | 11 | 13 | 1 | 0 |
| 13-03 | 4037 | 82 | 42 | 6 | 10 | 1 | 12 | 11 | 0 | 0 |
| 14-03 | 2938 | 97.4 | 51 | 11 | 5 | 9 | 9 | 11 | 1 | 0 |
| 15-03 | 1803 | 103 | 46 | 21 | 4 | 7 | 7 | 17 | 1 | 0 |
| 16-03 | 1322 | 102 | 54 | 17 | 2 | 11 | 0 | 16 | 2 | 0 |
| 17-03 | 3727 | 88 | 39 | 15 | 2 | 11 | 6 | 12 | 1 | 2 |
| 18-03 | 4633 | 104 | 33 | 27 | 5 | 11 | 13 | 14 | 1 | 0 |
| 19-03 | 3719 | 99.7 | 48 | 17 | 0 | 13 | 4 | 16 | 2 | 0 |
| 20-03 | 1940 | 97.3 | 50 | 16 | 0 | 7 | 7 | 16 | 0 | 1 |
| 21-03 | 2282 | 98 | 48 | 2 | 4 | 21 | 7 | 15 | 1 | 0 |
| 22-03 | 1437 | 101.7 | 48 | 19 | 0 | 9 | 10 | 16 | 0 | 0 |

**Afternoon: 12:00 to 6:00 p.m.**

| Date | Measured mass ppb C | Calculated mass % | Gasoline exhaust % | Diesel exhaust % | Evap % | LP gas % | Food cook. % | Asphalt % | Paint % |
|---|---|---|---|---|---|---|---|---|---|
| 23-02 | 876 | 103 | 56 | 21 | 1 | 10 | 4 | 11 | 0 |
| 24-02 | 758.7 | 109 | 44 | 40 | 1 | 5 | 8 | 11 | 0 |
| 25-02 | 1223.9 | 107 | 52 | 32 | 1 | 7 | 3 | 11 | 1 |
| 26-02 | 1125.1 | 107 | 47 | 38 | 2 | 1 | 6 | 14 | 0 |
| 27-02 | 857.1 | 109.3 | 48 | 37 | 2 | 1 | 9 | 14 | 0 |
| 28-02 | 1529 | 98.9 | 57 | 16 | 12 | 1 | 0 | 12 | 0 |
| 01-03 | 1643 | 103.3 | 56 | 11 | 10 | 5 | 5 | 13 | 0 |
| 02-03 | 741.6 | 101.6 | 51 | 23 | 2 | 9 | 8 | 11 | 0 |
| 03-03 | 848.3 | 104.5 | 47 | 34 | 1 | 7 | 4 | 12 | 0 |
| 04-03 | 1193.2 | 97.9 | 47 | 25 | 1 | 2 | 10 | 13 | 0 |
| 05-03 | 587.7 | 101.3 | 54 | 29 | 1 | 0 | 1 | 15 | 0 |
| 06-03 | 558 | 95.2 | 47 | 30 | 0 | 1 | 4 | 13 | 0 |
| 07-03 | 1076 | 107.6 | 49 | 42 | 0 | 3 | 1 | 13 | 0 |
| 08-03 | 644.4 | 98.7 | 40 | 36 | 0 | 3 | 4 | 14 | 0 |
| 09-03 | 1000.7 | 91.8 | 38 | 24 | 1 | 6 | 12 | 9 | 0 |
| 10-03 | 1942.3 | 105.2 | 44 | 29 | 7 | 2 | 11 | 12 | 0 |
| 11-03 | 1385.5 | 103 | 43 | 41 | 0 | 3 | 5 | 11 | 0 |
| 12-03 | 1005.7 | 102.8 | 42 | 41 | 0.3 | 0 | 9 | 11 | 0 |
| 13-03 | 1583.8 | 102.1 | 62 | 25 | 0.3 | 1 | 0 | 14 | 0 |
| 14-03 | 1006 | 105.6 | 40 | 46 | 0.2 | 0.4 | 7 | 12 | 0 |
| 15-03 | 785.6 | 87 | 39 | 28 | 0.2 | 3 | 7 | 11 | 0 |
| 16-03 | 926.5 | 101.8 | 42 | 31 | 0.8 | 6 | 12 | 11 | 0 |
| 17-03 | 2373.1 | 70.5 | 27 | 19 | 7 | 0 | 6 | 12 | 0 |
| 18-03 | 1838.8 | 102 | 48 | 23 | 7 | 2 | 9 | 14 | 0 |
| 19-03 | 2090.7 | 108.6 | 50 | 28 | 5 | 2 | 4 | 18 | 1 |
| 20-03 | 2351 | 102.1 | 50 | 21 | 3 | 2 | 10 | 15 | 0 |
| 21-03 | 706 | 97.8 | 59 | 19 | 0.1 | 2 | 1 | 15 | 0 |
| 22-03 | 1252.6 | 104.3 | 67 | 16 | 0 | 3 | 3 | 13 | 0 |

## Spatial Variation of NMHC Apportionment

La Merced station had the highest total NMHC average concentrations with 4,106 ppbC, followed by Xalostoc with 3,129 ppbC. The residential area of Pedregal was the least polluted area with 1,136 ppbC. In the past, the highest concentrations of NMHC had been measured in Xalostoc[10]. Figs. 4, 5, and 6 show the percentage contribution of measured NMHC of each source to the atmosphere, calculated using the CMB model, at three receptor sites (Xalostoc, La Merced, and Pedregal) for each date of the sampling period. Source contribution estimates had variations among the three sites, but in general, the major contributors to NMHC were vehicular exhaust emissions (gasoline and diesel), reaching a sum together of 78% of the total, followed by LP gas contributing 6 to 38%, asphalt operations from 6 to 16%, evaporative emissions from 1 to 13%, food cooking from 1 to 13%, and painting operations up to 2%.

In Xalostoc station, gasoline-powered vehicle exhaust was the main NMHC contribution source, with an average of 34 ± 7.5%. Diesel-powered vehicles contributed 16 ± 5.7% on average, although it is possible that part of this high emission had come from the boilers of this industrial area, which has an extensive use of diesel as a fuel. Xalostoc had the highest LP gas emissions of the three sites, with an average of 22 ± 8%, owing to the presence of several LP gas distributor industries in this area. Xalostoc also had the highest fuel evaporative emissions value of the three sites, with an average of 5 ± 4%, the cause of which could be the use of petroleum distillates in some industrial processes. Food cooking emissions were estimated at 5 ± 3%, asphalt emissions were 10 ± 2%, and painting emissions contributed less than 1%. The source denominated "others", which corresponds mainly to unidentified compounds, had an important contribution (10 ± 4%). This suggests that other sources, such as industries within the area of interest, may contribute to the atmosphere with emissions of NMHC.Source contribution estimates in La Merced station are presented in Fig. 4. Exhaust emissions are the main source of NMHC, with average values of 47 ± 7% and 11 ± 3% for gasoline- and diesel-powered vehicles, respectively. These results are similar to those obtained with the 6-h integrated morning samples. Evaporative emissions represented 3 ± 3% of the NMHC total concentration. The LP gas contribution was 15 ± 6% and the contribution of food cooking was 3 ± 1%. The average LP gas contribution was higher during the 3 h in the morning in comparison with the results from 6-h integrated samples. Asphalt operations contributed 9 ± 2% and painting operations contributed 2 ± 1%. The landfill source was apportioned only 5 days. The unexplained source "others" had a contribution of 5 ± 4%.

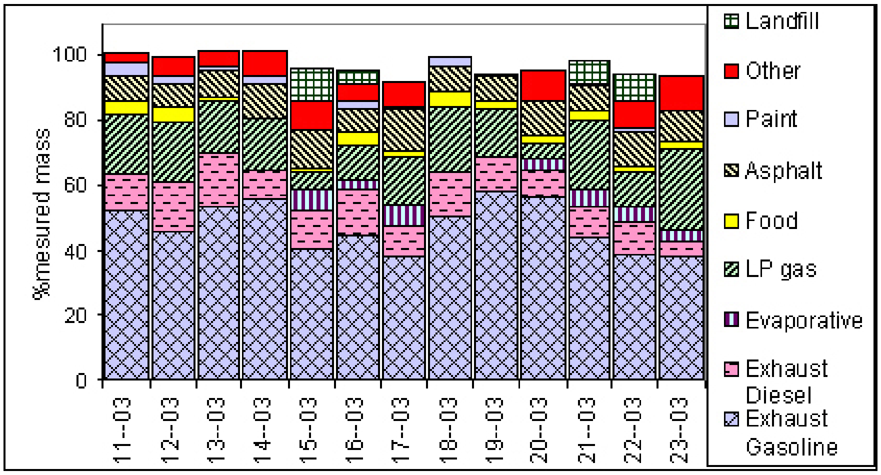

**FIGURE 4.** Average source contributions in La Merced.

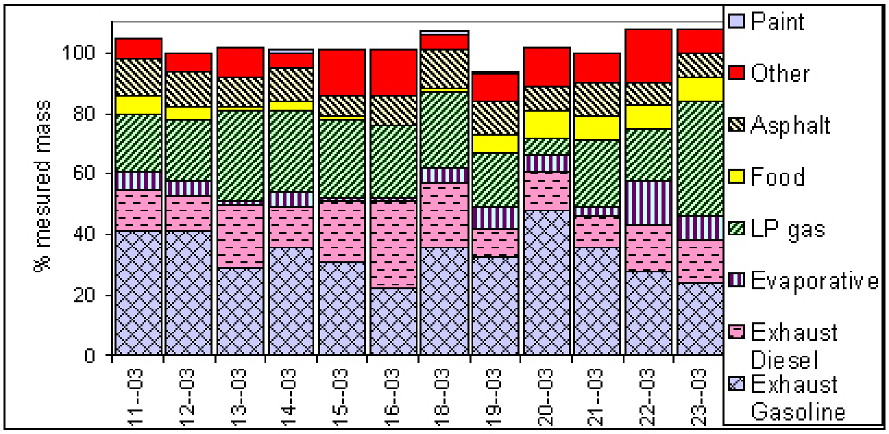

**FIGURE 5.** Average source contributions in Xalostoc.

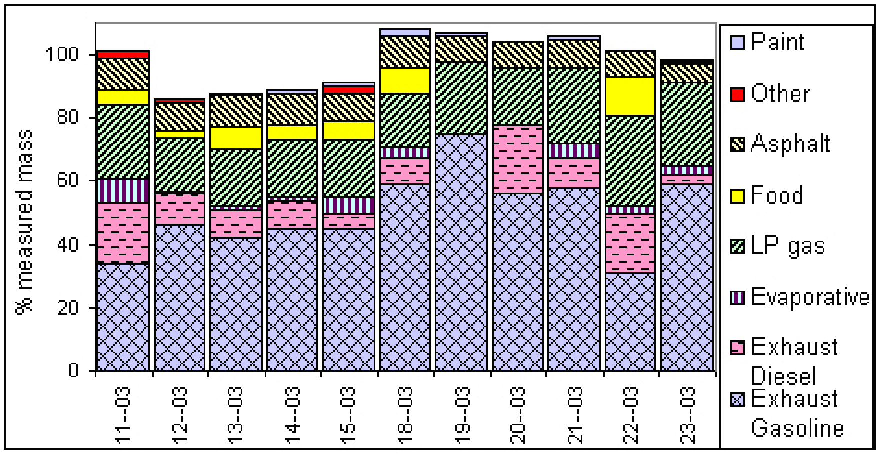

**FIGURE 6.** Average source contributions in Pedregal.

Pedregal was the least polluted area compared to Xalostoc and La Merced. Gasoline-powered vehicles were the main source of emissions, with average contributions of 50 ± 13%. Pedregal is a residential area and in general had a smaller contribution from diesel-powered vehicles than the other sites, with an average of 10 ± 7%. Evaporative emissions contributed an average of 2.7 ± 3%. The LP gas emissions had an average of 21 ± 4%, and food cooking contributed 6 ± 2%. The contribution of asphalt operations averaged 9 ± 1%, and painting emissions reached up to 2%.

## Individual NMHC Apportionment

Source contributions to the presence of individual NMHC were also determined with the CMB model. The major source of most of the organic species was the vehicular source, with some exceptions. Tables 4, 5, and 6 show the source contributions to the emissions of the ten most abundant NMHC species at different receptor sites. Motor vehicle exhaust emissions (gasoline and diesel) were the major contributors of acetylene, ethylene, *iso*-pentane, *n*-hexane, *n*-pentane, toluene, and *o*-,*m*-, and *p*-xylenes. The most important source of propane, butane, and *iso*-butane was the handling and distribution of LP gas (>50%), followed by vehicular emissions. On the

**TABLE 4**
**Individual NMHC Source Apportionment in La Merced**

| Species | Exhaust, gasoline | Exhaust, diesel | Evaporative | Asphalt | LP gas | Food | Paint | Landfill |
|---|---|---|---|---|---|---|---|---|
| **Ethylene** | 69.6 ± 9.2 | 10.9 ± 3.8 | 0.0 ± 0.0 | 9.7 ± 2.9 | 0.0 ± 0.0 | 9.5 ± 6.8 | 0.0 ± 0.0 | 0.3 ± 0.1 |
| **Acethylene** | 82.1 ± 4.9 | 6.4 ± 2.7 | 0.0 ± 0.0 | 7.3 ± 2 | 0.0 ± 0.0 | 3.6 ± 2.7 | 0.0 ± 0.0 | 0.51 ± 0.5 |
| **Propane** | 13.5 ± 5.2 | 6.8 ± 3.1 | 0.0 ± 0.0 | 9.1 ± 4.8 | 64.3 ± 10.4 | 5.2 ± 3.8 | 0.0 ± 0.0 | 0.9 ± 0.8 |
| **Iso-butane** | 17 ± 5.8 | 9.5 ± 4 | 1.4 ± 1.3 | 9.9 ± 4.7 | 56 ± 11.5 | 5.1 ± 3.7 | 0.0 ± 0.0 | 1 ± 1 |
| **Iso-pentane** | 62.5 ± 13.7 | 18.5 ± 6.6 | 13.6 ± 11.0 | 3.1 ± 1.2 | 0.0 ± 0.0 | 0.0 ± 0.0 | 1.5 ± 0.8 | 1 ± 1 |
| **n-Butane** | 21.7 ± 10.9 | 7.5 ± 3.3 | 4.2 ± 2.6 | 7.6 ± 2.8 | 52.4 ± 18.5 | 5.3 ± 3.8 | 0.0 ± 0.0 | 1 ± 1 |
| **n-Hexane** | 55.1 ± 9 | 18.9 ± 6.5 | 9.6 ± 6.3 | 11.9 ± 2.6 | 0.0 ± 0.0 | 0.2 ± 0.2 | 3.3 ± 3 | 1.2 ± 1 |
| **n-Pentane** | 58.1 ± 15.3 | 12.1 ± 5.4 | 19.4 ± 16.5 | 6.4 ± 1.7 | 0.0 ± 0.0 | 0.0 ± 0.0 | 2.5 ± 1.9 | 1.3 ± 1.2 |
| **Toluene** | 59.7 ± 6.6 | 19.3 ± 5.3 | 1.5 ± 1.2 | 11.8 ± 3.1 | 0.0 ± 0.0 | 0.9 ± 0.3 | 5.1 ± 3.1 | 1.3 ± 1 |
| **Xylenes** | 43.9 ± 8.6 | 23.6 ± 7 | 1.0 ± 0.6 | 23 ± 5.5 | 0.0 ± 0.0 | 0.0 ± 0.0 | 1.1 ± 0.8 | 6.3 ± 2.4 |

**TABLE 5**
**Individual NMHC Source Apportionment in Xalostoc**

| Species | Exhaust, gasoline | Exhaust, diesel | Evaporative | Asphalt | LP gas | Food | Paint |
|---|---|---|---|---|---|---|---|
| **Ethylene** | 58.5 ± 6.2 | 15.5 ± 6 | 0.3 ± 1.1 | 11.8 ± 3.2 | 0.0 ± 0.0 | 13.8 ± 7.9 | 0.0 ± 0.0 |
| **Acethylene** | 74.8 ± 5.4 | 9.5 ± 3.2 | 0.2 ± 0.7 | 9.5 ± 2.7 | 0.0 ± 0.0 | 5.9 ± 3.5 | 0.0 ± 0.0 |
| **Propane** | 6.1 ± 2.7 | 5.0 ± 2.3 | 0.0 ± 0.0 | 5.5 ± 1.7 | 78.5 ± 7.7 | 4.6 ± 2.9 | 0.0 ± 0.0 |
| **Iso-butane** | 8.5 ± 3.4 | 7.6 ± 3.4 | 1.6 ± 1.1 | 6.7 ± 1.8 | 70.8 ± 9 | 4.9 ± 2.9 | 0.0 ± 0.0 |
| **Iso-pentane** | 45.6 ± 13.2 | 22.4 ± 9.6 | 26.2 ± 13.8 | 3.6 ± 1 | 0.0 ± 0.0 | 0.0 ± 0.0 | 2 ± 1.1 |
| **n-Butane** | 14.5 ± 5.5 | 8.6 ± 3.8 | 3.2 ± 1.7 | 7.8 ± 1.9 | 58.3 ± 11.4 | 7 ± 4.1 | 0.0 ± 0.0 |
| **n-Hexane** | 41.7 ± 9.7 | 22.7 ± 8 | 18.1 ± 8.7 | 12.7 ± 2.4 | 0.0 ± 0.0 | 0.0 ± 0.0 | 4.7 ± 3.6 |
| **n-Pentane** | 42.4 ± 14 | 13.4 ± 4.7 | 34.5 ± 19 | 6.9 ± 2.1 | 0.0 ± 0.0 | 0.2 ± 0.3 | 2.2 ± 1.3 |
| **Toluene** | 46.8 ± 8.3 | 25.5 ± 7.7 | 5.4 ± 4.6 | 14.3 ± 2.6 | 0.0 ± 0.0 | 1.3 ± 0.7 | 6.5 ± 4.2 |
| **Xylenes** | 34.4 ± 6.6 | 30.9 ± 7.9 | 4.2 ± 6.4 | 28.3 ± 5.2 | 0.0 ± 0.0 | 1.3 ± 1.2 | 0.8 ± 1 |

**TABLE 6**
**Individual NMHC Source Apportionment in Pedregal**

| Species | Exhaust, gasoline | Exhaust, diesel | Evaporative | Asphalt | LP gas | Food | Paint |
|---|---|---|---|---|---|---|---|
| **Ethylene** | 68.3 ± 18.4 | 9.3 ± 5.2 | 0.1 ± 0.2 | 8.3 ± 2.0 | 0.0 ± 0.0 | 13.9 ± 10.7 | 0.0 ± 0.0 |
| **Acethylene** | 81.2 ± 11.7 | 5.5 ± 3.6 | 0.0 ± 0.0 | 6.5 ± 2.3 | 0.0 ± 0.0 | 6.6 ± 5.1 | 0.0 ± 0.0 |
| **Propane** | 9.4 ± 3.1 | 3.8 ± 2.2 | 0.0 ± 0.0 | 5.2 ± 1.2 | 75 ± 6.5 | 6.2 ± 5.6 | 0.0 ± 0.0 |
| **Iso-butane** | 13 ± 3.7 | 5.7 ± 3.2 | 0.7 ± 0.5 | 6.3 ± 1.5 | 68.2 ± 6.5 | 6.4 ± 3.1 | 0.0 ± 0.0 |
| **Iso-pentane** | 66.2 ± 16.7 | 17.1 ± 9.8 | 11.3 ± 8.8 | 3.1 ± 0.9 | 0.0 ± 0.0 | 0.0 ± 0.0 | 2.3 ± 2.9 |
| **n-Butane** | 5.5 ± 6.3 | 5.4 ± 3.1 | 1.1 ± 0.9 | 6 ± 2.1 | 62.8 ± 9.6 | 7.3 ± 5.7 | 0.0 ± 0.0 |
| **n-Hexane** | 61.1 ± 16.1 | 16.9 ± 5.6 | 7.7 ± 11.9 | 11.6 ± 2.9 | 0.0 ± 0.0 | 0.0 ± 0.0 | 2.6 ± 1.7 |
| **n-Pentane** | 65 ± 17 | 11 ± 5.6 | 16.3 ± 12 | 6.6 ± 1.9 | 0.0 ± 0.0 | 0.1 ± 0.1 | 1.8 ± 1.7 |
| **Toluene** | 57.9 ± 14.7 | 16.3 ± 9.5 | 1.8 ± 1.5 | 10.9 ± 2.8 | 0.0 ± 0.0 | 1.6 ± 1.2 | 11.4 ± 8.6 |
| **Xylenes** | 49.3 ± 14.4 | 22 ± 11.1 | 1.5 ± 1.3 | 24.4 ± 4.7 | 0.0 ± 0.0 | 1.5 ± 1.6 | 1.2 ± 1.2 |

other hand, food cooking contributed to the presence in the atmosphere of acetylene, ethylene, propane, isobutane, and butane, with averages of 5, 12, 5, 6, and 7%, respectively. The presence of two carbon compounds in ambient air has always been attributed to vehicle sources, but the results obtained in this study suggest that food cooking is also a source of these compounds.

Asphalt operations and landfill emissions were contributors of xylenes with averages of 25 and 6%, respectively. Painting operations also contributed to the presence of toluene, *n*-hexane, and *n*-pentane (6, 4, and 2%, respectively). The evaporative vehicular emissions made an important contribution to the presence of *iso*- and *n*-pentane, although the high percentage of gasoline vapors in the industrial site (Xalostoc) suggests that there is another source emitting these compounds, for instance, the opened tanks of some petroleum distillates used in industrial processes.

## Comparison With the Emission Inventory

The comparison between CMB results and emission inventories is limited essentially because the emission inventory estimates represent a 24-h emissions average for the whole year, while the data used in CMB calculations represent limited days and hours. However, several studies have been carried out to relate receptor model estimates to emission inventories. These studies have established that the CMB results provide a sample-specific estimate of the portion of the emission inventory that has contributed to the particular NMHC mass being sampled. When enough samples are collected, the average value of CMB coefficients is an estimate of the overall emission inventory[3,7,8].

In order to make a comparison between the emission inventory and CMB results obtained in the present study, the fractional contributions obtained from CMB were adjusted by removing the contributions of food cooking emissions, because the inventory has not included such source. Fig. 7 shows the comparison of CMB estimates and the MCMA emission inventory. The average source contribution at each site was obtained for each site and plotted. The average gasoline vehicular emissions estimated with the CMB model showed similarities compared to those reported in the inventory, although the emissions estimated using the model in Xalostoc were somewhat lower than the emission inventory predictions. On the other hand, diesel exhaust contribution calculated by the model was higher than those values reported in the inventory by a factor of two. This result suggests an underestimation of this source in the inventory. The difference between the evaporative emissions of the inventory (2%) and the CMB model estimates (4.6%) is due to the fact that evaporative emissions estimated in the inventory include only the distribution and storage of fuels, while CMB model estimates also include hot soak emissions. The average contributions of LP gas and painting and graphic arts emissions obtained when applying the model were quite similar to those reported in the inventory. It should be remarked that the CMB estimates for asphalt processes were very consistent and five orders of magnitude higher than the emissions reported by the inventory. This implies an underestimation of asphalt operations in the emission inventory.

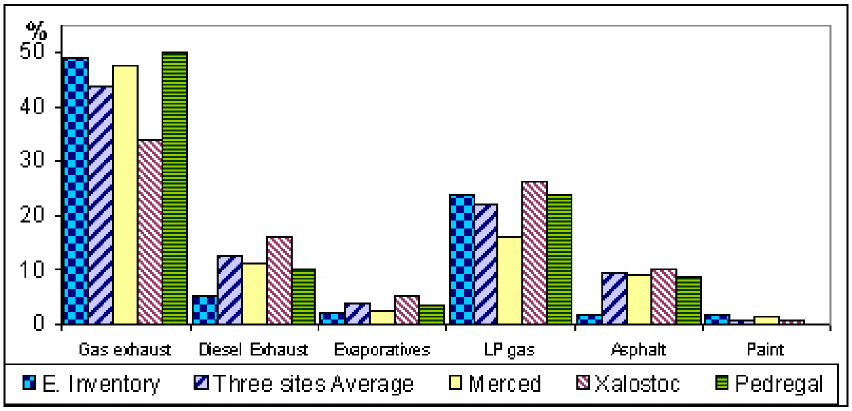

**FIGURE 7.** Comparison of CMB and emission inventory estimates.

## CONCLUSIONS

NMHC source profiles for vehicular exhaust and food cooking emissions were determined in the MCMA in order to apply the CMB model to ambient data obtained at different times and at three sites in an intensive sampling campaign carried out during 1997. The average NMHC concentrations found were 4,106 ppbC in the commercial area, 3,129 ppbC in the industrial area, and 1,136 ppbC in the residential district. The results obtained showed that the concentrations of NMHC were higher during the morning and lower during the afternoon. Throughout the day the NMHC concentrations decreased due to an improved dispersion and as a result of photochemical reactions generated in the atmosphere when temperature and solar radiation increased.The model performance measures were individually examined for each CMB fit, and in all cases were within the acceptable intervals. The major contributions to NMHC were light- and heavy-duty vehicular exhausts for all sites during the sampling period, followed by handling and distribution of LP gas. The asphalt emissions, food cooking emissions, painting-operations emissions, and landfill emissions contributed in a smaller proportion. The average of both gasoline and diesel exhaust increased during the day from 59 to 77% mainly because of diesel-powered vehicle activity. On the other hand, favorable dispersion conditions during the afternoon promoted the decrease of the evaporative emissions from 5.7 to 2.4%, as well as the emissions from handling and distribution of LP gas, which had an average value of 12% in the morning and 4% in the afternoon. Contributions of other sources had similar values in both periods with 6 and 7% for food cooking, 14 and 12% for asphalt processes, and around 1% for painting operations.

Source contributions showed variations among the different sampling sites according to the land use. The La Merced 3-h integrated sample apportionment showed some differences compared to the 6-h integrated morning samples: the vehicle exhaust contribution averaged 58% (gasoline and diesel); LP gas, 15%; food cooking, 3%; asphalt operations, 9%; and painting operations, 1%. Landfill emissions contributed 3% in this location. In Xalostoc, source contributions were: vehicle exhaust, 50% (gasoline- and diesel-powered vehicles); LP gas, 22%; evaporative emissions, 5%; food cooking, 5%; asphalt operations, 10%; and painting operations, 1%. The contribution of the unexplained source named “others” was significant in Xalostoc (10%) and La Merced (6%), perhaps due to industrial or service emissions not considered in this study. Pedregal was the least polluted area, with an average contribution from vehicular emissions of 59% (gasoline and diesel powered-vehicles); LP gas, 21%; food cooking, 6%; evaporative emissions, 2.7%; asphalt operations, 9%; and painting operations, 1%. Apportionment of individual NMHC species showed that the major contribution source of the majority of the compounds is motor vehicle exhaust, except for propane, *n*-butane, and *iso*-butane, which are emitted mainly during handling and distribution of LP gas. Gasoline vapors are important contributors of *n*- and *iso*-pentane. Asphalting operations, vehicular sources, and landfills emit xylenes as well as toluene, although painting operations contribute to the presence of this aromatic compound with an average of 6%. Source contribution estimates were compared to the Emission Inventory for MCMA. Agreement of the CMB model results with the Emission Inventory was good for gasoline-powered vehicle exhaust, handling and distribution of LP gas, and painting operations. The estimated contribution of NMHC from food cooking showed that this source should be considered in the Emission Inventory. The asphalt emissions estimated by the CMB model were almost fivefold higher than those reported in the inventory. Diesel contribution calculated by the model was two orders higher than the values reported by the inventory. These results suggest an underestimation of asphalt and diesel emissions that should be reviewed in order to apply adequate strategies of NMHC control in the MCMA.

## ACKNOWLEDGEMENTS

PEMEX and IMP supported this research as part of the project FIES-04-VI.
